# Accuracy of the estimations of respiratory mechanics using an expiratory time constant in passive and active breathing conditions: a bench study

**DOI:** 10.1186/s40001-023-01146-y

**Published:** 2023-06-24

**Authors:** Yuqing Chen, Yueyang Yuan, Hai Zhang, Feng Li

**Affiliations:** 1 Department of Respiratory Medicine, Shanghai Chest Hospital, Shanghai Jiao Tong University, Shanghai, 200030 China; 2 School of Mechanical and Electrical Engineering, Hu Nan City University, Yi Yang, 413099 China

**Keywords:** Mechanical ventilation, Respiratory mechanics, Lung compliance, Airway resistance

## Abstract

**Background:**

Respiratory mechanics monitoring provides useful information for guiding mechanical ventilation, but many measuring methods are inappropriate for awake patients. This study aimed to evaluate the accuracy of dynamic mechanics estimation using expiratory time constant (RC_exp_) calculation during noninvasive pressure support ventilation (PSV) with air leak in different lung models.

**Methods:**

A Respironics V60 ventilator was connected to an active breathing simulator for modeling five profiles: normal adult, restrictive, mildly and severely obstructive, and mixed obstructive/restrictive. Inspiratory pressure support was adjusted to maintain tidal volumes (V_T_), achieving 5.0, 7.0, and 10.0 ml/kg body weight. PEEP was set at 5 cmH_2_O, and the back-up rate was 10 bpm. Measurements were conducted at system leaks of 25–28 L/min. RC_exp_ was estimated from the ratio at 75% exhaled V_T_ and flow rate, which was then used to determine respiratory system compliance (C_rs_) and airway resistance (R_aw_).

**Results:**

In non-obstructive conditions (R_aw_ ≤ 10 cmH_2_O/L/s), the C_rs_ was overestimated in the PSV mode. Peak inspiratory and expiratory flow and V_T_ increased with PS levels, as calculated C_rs_ decreased. In passive breathing, the difference of C_rs_ between different V_T_ was no significant. Underestimations of inspiratory resistance and expiratory resistance were observed at V_T_ of 5.0 ml/kg. The difference was minimal at V_T_ of 7.0 ml/kg. During non-invasive PSV, the estimation of airway resistance with the RC_exp_ method was accurately at V_T_ of 7.0 ml/kg.

**Conclusions:**

The difference between the calculated C_rs_ and the preset value was influenced by the volume, status and inspiratory effort in spontaneously breathing.

## Background

Mechanical ventilation is a lifesaving intervention that has become widely used in the management of critically ill patients [1, 2]. The exact settings of the ventilator must be adequately adjusted according to the patients’ conditions to optimize the patient outcomes and prevent ventilator-induced injury and complications [2, 3]. The analysis of individual respiratory mechanics is beneficial for guiding the ventilator setting under the conditions of lung-protective mechanical ventilation [4].

In the past 15 years, the focus on respiratory mechanics analysis changed from the static to the dynamic conditions [5]. “Static” or “quasi-static” conditions mean that the analysis of the respiratory mechanics is performed under conditions of zero airflow, which is typically carried out using an end-inspiration and an end-expiration pause [6]. “Dynamic” conditions mean that the measurement is performed under conditions of no flow interruption during mechanical ventilation [7]. The advantage of the dynamic analysis is that respiratory maneuvers such as zero-flow occlusion and the interruption of the patient spontaneous breathing are not required.

Non-invasive positive pressure ventilation (NPPV) is used in patients with mild to moderate respiratory failure, since relatively stable spontaneous breathing is necessary, and air leaks are always present when using a face mask [8]. Pressure support ventilation (PSV) is one of the most used modes of non-invasive ventilation, which also requires the patient’s breathing effort to trigger. Despite the wide use of PSV, the accurate estimation of the respiratory mechanics during PSV is still a problem, and the static methods are applied to PSV, since airflow is always present and variable both in inspiration and expiration [9, 10].

Recently, some dynamic approaches have been introduced, considerably refined by the improvement of static measurement, and addressing the need for accurate estimation of lung mechanics [11, 12]. One of the new continuous methods is based on estimating respiratory system compliance (C_rs_) and airway resistance (R_aw_), which do not depend on end-inspiratory occlusion. Al-Rawas et al. [13] proposed the expiratory time constant (RC_exp_) for the determination of C_rs_ and total R_aw_. RC_exp_ contains information about the mechanical properties of the respiratory system, but it still refinement, particularly under the condition of air leaks.

We hypothesized that real-time sampling of the respiratory data obtained from the inspiration and expiration phases would improve the precision of the estimation of the respiratory system mechanics. Hence, the purpose of this bench study was to evaluate the accuracy of dynamic mechanics estimation using RC_exp_ calculation during PSV with air leak in different lung models.

## Methods

### Lung models

The ASL 5000 Breathing Simulator (IngMar Medical, Pittsburg, PA, USA) features a computerized lung simulator comprising a piston moving in a cylinder. The simulator is a single-compartment model [14]. Respiratory mechanics conditions were adjusted to simulate an adult patient (65–70 kg body weight) placed in the semi-recumbent position (inclination of 45°). Five respiratory mechanics conditions were preset: mildly obstructive (R_aw_ = 10 cmH_2_O/L/s, static C_rs_ = 50 mL/cmH_2_O, and rate = 15 breaths/min); severely obstructive (R_aw_ = 20 cmH_2_O/L/s, static C_rs_ = 50 mL/cmH_2_O, and rate = 15 breaths/min); restrictive (R_aw_ = 10 cmH_2_O/L/s, static C_rs_ = 25 mL/cmH_2_O, and rate = 30 breaths/min); mixed obstructive and restrictive (R_aw_ = 20 cmH_2_O/L/s, static C_rs_ = 25 mL/cmH_2_O, and rate = 15 breaths/min); and normal adult (R_aw_ = 5 cmH_2_O/L/s, static C_rs_ = 50 mL/cmH_2_O, and rate = 15 breaths/min). The inspiratory time was set at 0.8 s for the restrictive conditions and 1.6 s for the other conditions [15-17]. The patient’s inspiratory effort was −5 cmH_2_O for the normal, obstructive, and mixed obstructive/restrictive conditions and −10 cmH_2_O for the restrictive condition. Pressure reduction produced 300 ms following initiation of an obstructed inspiratory effort was −3.6 cmH_2_O. A semi-sinusoidal inspiratory waveform was chosen, with the rise and release times each of 50%, and an inspiratory hold time of 0%. The simulator integrates user-controlled leaks using a plateau exhalation valve (PEV). In the current study, air leak was controlled between 24 and 26 L/min with 20 cmH_2_O peak airway pressure [18]. The inspired oxygen fraction (F_I_O_2_) was maintained at 0.21.

The patient–mask interface was simulated using a mannequin head. Endotracheal tubes (inner diameter, 22 mm) placed in the mouth and nostrils directed the gas from the facemask to the simulator. An oro-nasal facemask without exhalation ports (BestFit^™^; Curative Medical Inc., Santa Clara, CA, USA) was fastened firmly to the mannequin head using standard straps. A leak flow below 1–2 L/min was obtained at 20 cmH_2_O positive pressure after PEV removal [20].

### Ventilator settings

This bench study was performed using a dry circuit without a humidifier. First, five passive conditions with zero breathing frequency and zero inspiratory muscle pressure (P_mus_) were simulated. A Hamilton C3 ventilator (Hamilton Medical AG, Bonaduz, Switzerland) was linked to the lung simulator without facemask and PEV. The ventilator was calibrated and configured in the volume-controlled ventilation (VCV) mode. Then, it was configured in the pressure-controlled ventilation (PCV) mode. Finally, active conditions with a spontaneous effort were simulated. A Respironics V60 Bilevel Ventilator (Philips, Best, The Netherlands) was also connected to the lung simulator via a 1.8-m-long single-use, single limb, corrugated circuit with facemask and PEV. The V60 ventilator was calibrated and configured in the PSV mode. The ventilator’s parameters were set according to respiratory mechanics profiles: positive end-expiratory pressure (PEEP). The PC and PS levels were adjusted to obtain tidal volumes (V_T_), achieving 5.0, 7.0, and 10.0 ml/kg body weight outputted by the ventilator using a back-up respiratory rate of 10 breaths/min and maximal duration of the inspiratory phase of 2.0 s. The shorter inspiratory rise time was selected but avoiding overshoot [19, 20]. The trigger sensitivity and cycling criteria were auto-adjusted in the PSV mode (digital Auto-Trak^™^) [21].

### Data collection

After baseline pressure stabilization, air leaks from the PEV were supplemented to the system, with  ≥ 5 min allowed for ventilator/simulator synchronization. In the case of synchronization failure, sensitivity, and/or inspiratory effort were changed. If synchronization remained unachievable, the ventilator was regarded as unfit for assisted ventilation at that level of the leak. Upon stabilization, eight breaths were recorded at 1-min intervals. The offline assessment of all breaths was carried out with the software provided with the ASL 5000 Breathing Simulator.

In the inspiratory phase, peak inspiratory flow (PIF), end-inspiration pressure (EIP), inspiratory time (T_I_), expiratory tidal volume (V_TE_) were measured by the simulator. The peak expiratory flow (PEF) and total PEEP were sampled in the expiration phase.

The RC_exp_ was estimated by the ratio between volume and flow at 75% of the expiratory V_T_ (TEF75) [22]. The quasi-static two-point compliance of the respiratory system (C_rs_) was calculated as the ratio between V_TE_ and driving pressure (ΔP). The driving pressure was calculated as the difference between EIP and total PEEP, measured at end-inspiration and end-expiration, respectively. Because subjects were ventilated in the pressure support mode (with exponential decay of inspiratory flow waveform), the inspiratory resistance (R_insp_) was estimated using Eq. 3, and the expiratory resistance (R_exp_) was calculated using Eq. 4 [13, 23]:

1 $${\text{C}}_{{{\text{rs}}}} \, = \,{\text{V}}_{{{\text{TE}}}} \,/\,\left( {{\text{EIP}}\, - \,{\text{PEEP}}} \right)$$

2 $${\text{RC}}_{{{\text{exp}}}} \, = \,{75}\% {\text{V}}_{{{\text{TE}}}} \,/\,{\text{TEF75}}$$

3 $${\text{R}}_{{{\text{insp}}}} \, = \,\left( {{\text{EIP}}\, - \,{\text{PEEP}}} \right)\,/\,\left( {{\text{PIF}}\, + \,{\text{V}}_{{{\text{TE}}}} \,/\,{\text{RC}}_{{{\text{exp}}}} } \right)$$

4 $${\text{R}}_{{{\text{exp}}}} \, = \,{\text{RC}}_{{{\text{exp}}}} \,/\,{\text{C}}_{{{\text{rs}}}}$$

### Statistical analysis

Continuous data were presented as means ± standard deviations. The normality of the data was assessed by the Shapiro–Wilk test. Comparisons of variables at different settings were performed by one-way randomized block ANOVA. Statistical analysis was carried out with SPSS 19.0 (IBM, Armonk, NY, USA). Two-tailed *P* < 0.01 was considered statistically significant. Differences between calculated values with RC_exp_ method and preset values were expressed as the percentages of preset values. The smaller the error, the more clinically significant the parameter. The purpose of this study was to observe the error size, which should be optimally  ≤ 10%.

## Results

### Measured airflow and airway pressure at different VT ventilation in the various models

**Fig. 1:**
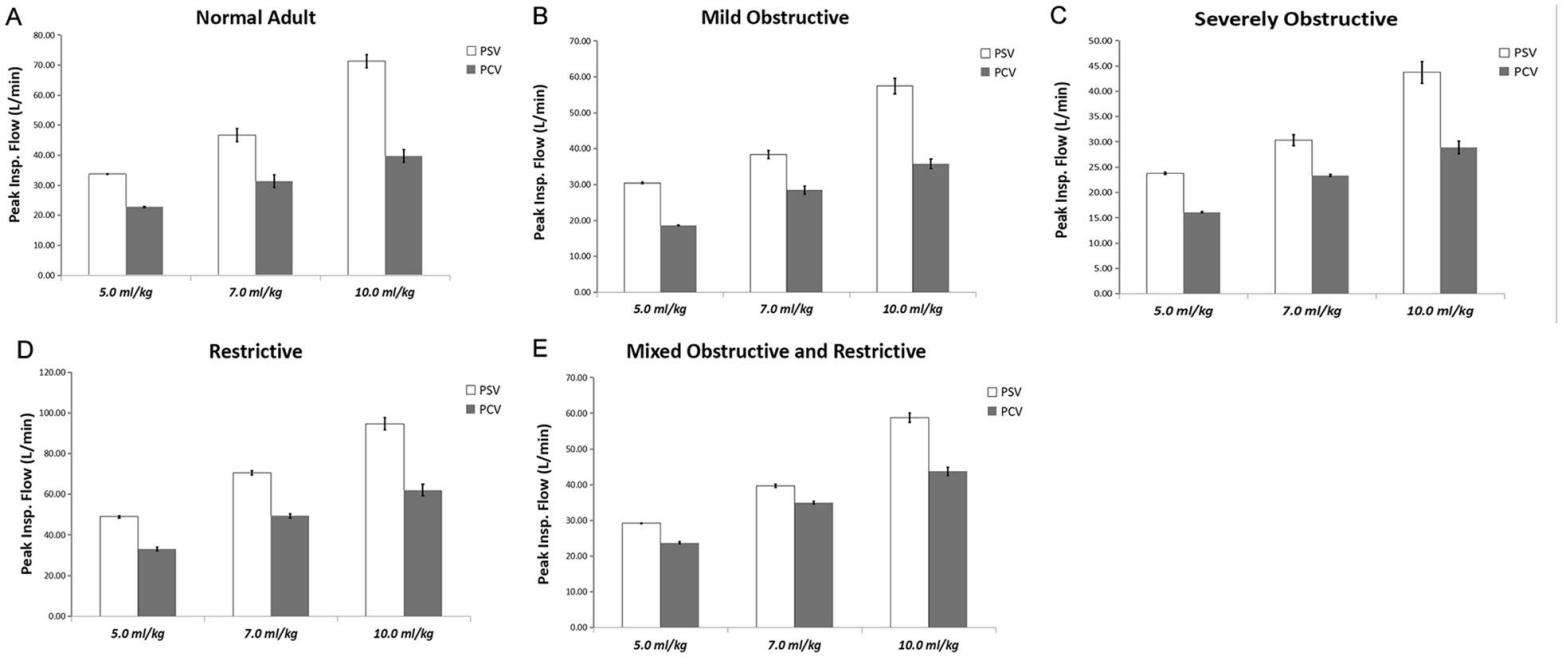
Comparisons of peak inspiratory flow (PIF) in various models during controlled and assisted ventilatory mode. Normal adult (**A**), mildly obstructive (**B**), severely obstructive (**C**), restrictive (**D**), and mixed (**E**) models. Data are presented as mean ± SD. *P* < 0.01 vs. PSV for all pairwise comparisons

**Fig. 2:**
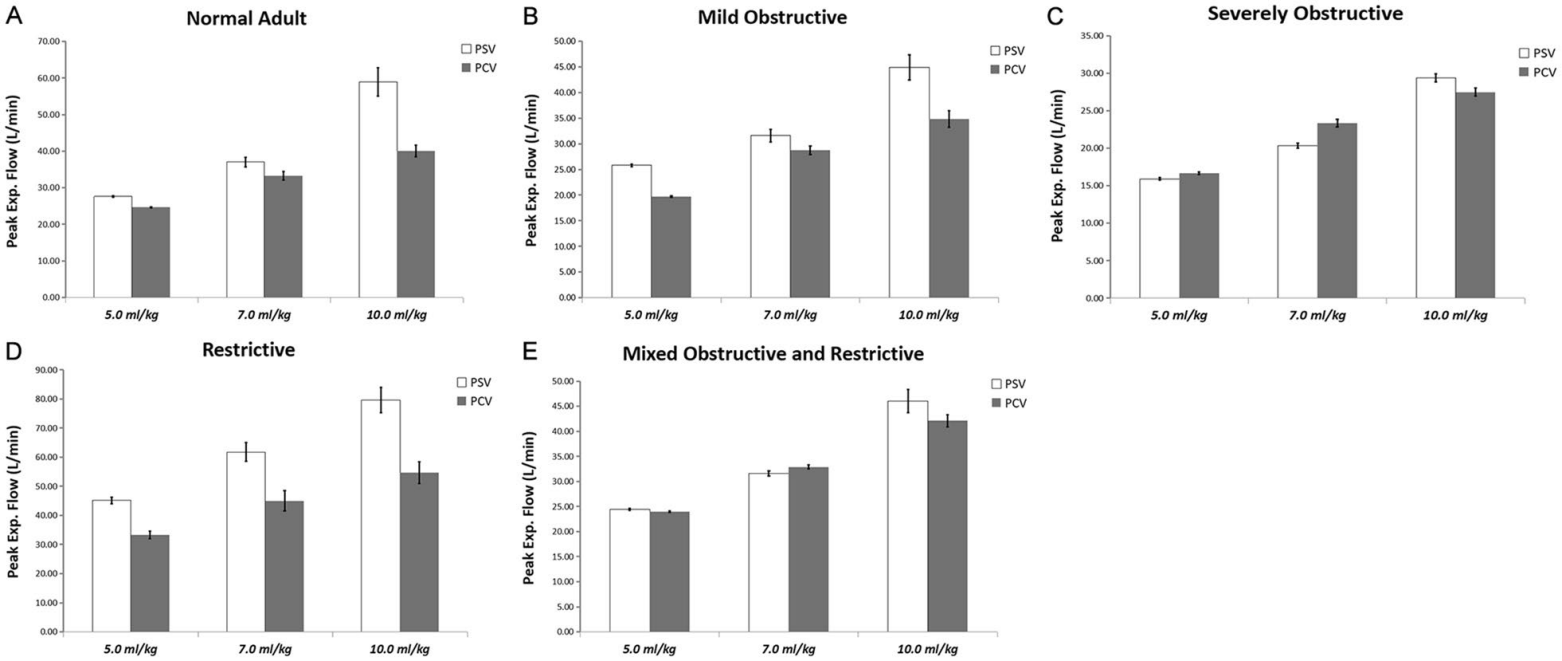
Comparisons of peak expiratory flow (PEF) in various models during controlled and assisted ventilatory mode. Normal adult (**A**), mildly obstructive (**B**), severely obstructive (**C**), restrictive (**D**), and mixed (**E**) models. Data are presented as mean ± SD. *P* < 0.01 vs. PSV for all pairwise comparisons

The results of the dynamic mechanics at different V_T_ levels are summarized in Tables 1, 2, 3, 4 and 5. The V60 ventilator was able to adapt to the system leak (25–28 L/min) without adjustment. Increasing the PS and PC levels was associated with higher PIF and PEF and larger tidal volume. In all lung models, PIF was always higher than PEF in the PSV mode. PIF in the PSV mode was also higher than in the PCV model (Figs. 1 and 2). Compared with passive breathing, the driving pressure was much lower than in active breathing conditions at V_T_ of 5.0 to 10.0 ml/kg.

**Table 1:** Comparison between VCV (test), PCV, and PSV in the normal adult lung model

	ΔP	PIF	PEF	C_rs_	RC_exp_	R_insp_	R_exp_
	(cmH2O)	(L/min)	(L/min)	(ml/cmH_2_O)	(ms)	(cmH_2_O/L·s)	(cmH_2_O/L·s)
VCV	8.80 ± 0.03	30.13 ± 0.58	49.72 ± 0.73	50.83 ± 1.31	541.96 ± 7.74	4.74 ± 0.54	10.66 ± 0.15
7.0 ml/kg							
PCV	6.16 ± 0.16	22.76 ± 0.13	24.65 ± 0.13	51.33 ± 1.39*	590.33 ± 6.82	16.21 ± 0.43	11.51 ± 0.32
5.0 ml/kg							
PCV	8.42 ± 0.05	31.31 ± 2.12	33.26 ± 1.22	51.66 ± 1.34	618.36 ± 7.11	16.11 ± 0.12	11.97 ± 0.16
7.0 ml/kg							
PCV	12.44 ± 0.06	39.70 ± 2.11	40.10 ± 1.61	49.31 ± 0.77	725.19 ± 7.65	18.78 ± 0.54	14.71 ± 0.13
10.0 ml/kg							
PSV	2.13 ± 0.07	33.70 ± 0.16	27.61 ± 0.22	152.44 ± 5.07	574.21 ± 4.30	3.78 ± 0.12	3.77 ± 0.13
5.0 ml/kg							
PSV	4.16 ± 0.05	46.63 ± 2.17	37.0641.34	100.23 ± 1.36	508.62 ± 6.48	5.35 ± 0.08^*^	5.07 ± 0.05
7.0 ml/kg						(*P* = 0.007)	
PSV	8.18 ± 0.09	71.34 ± 2.17	58.97 ± 3.87	74.26 ± 0.84	465.96 ± 4.36	6.87 ± 0.08	6.28 ± 0.08
10.0 ml/kg							
*F*	13,152.430	31,222.510	9316.271	2748.197	1414.282	4396.553	4846.627
*ANOVA p-value*	< 0.0001	< 0.000	< 0.0001	< 0.0001	< 0.0001	< 0.0001	< 0.0001

*V*_*TE*_ expiratory tidal volume, *N.S* not significant, *ΔP* driving pressure, *PIF* peak inspiratory flow, *PEF* peak expiratory flow, *C*_*rs*_ respiratory system compliance, *RC*_*exp*_ expiratory time constant, *R*_*insp*_ inspiratory airway resistance, *R*_*exp*_ expiratory airway resistance

*P values (Student *t* test) are for comparisons between PSV, PCV, and VCV. Data are shown as means ± standard deviation and are the results of eight measurements/case

**Table 2:** Comparisons between VCV (test), PCV, and PSV in the mildly obstructive lung model

	ΔP	PIF	PEF	C_rs_	RC_exp_	R_insp_	R_exp_
	(cmH_2_O)	(L/min)	(L/min)	(ml/cmH_2_O)	(ms)	(cmH_2_O/L·s)	(cmH_2_O/L·s)
VCV	8.99 ± 0.02	29.12 ± 0.05	41.76 ± 0.46	49.80 ± 0.38	446.50 ± 29.94	8.99 ± 0.56	12.92 ± 0.17
7.0 ml/kg							
PCV	6.37 ± 0.15	18.61 ± 0.11	19.70 ± 0.15	47.40 ± 1.36	531.02 ± 8.45	20.49 ± 0.11	11.20 ± 0.13
5.0 ml/kg							
PCV	9.34 ± 0.22	28.49 ± 1.13	28.79 ± 0.85	47.24 ± 1.25	533.90 ± 15.02	21.65 ± 0.12	11.30 ± 0.30
7.0 ml/kg							
PCV	13.39 ± 0.34	35.81 ± 1.36	34.86 ± 1.62	47.23 ± 0.75	544.10 ± 15.52	22.39 ± 0.25	11.52 ± 0.14
10.0 ml/kg							
PSV	3.94 ± 0.21	30.45 ± 0.25	25.81 ± 0.26	81.73 ± 4.20	586.49 ± 11.95	7.75 ± 0.44	7.19 ± 0.39
5.0 ml/kg							
PSV	6.09 ± 0.41	38.38 ± 1.15	31.60 ± 1.25	70.27 ± 1.37	620.37 ± 9.19	9.51 ± 0.16^*^	8.83 ± 0.13
7.0 ml/kg						(*P* = 0.025)	
PSV	11.11 ± 0.73	57.46 ± 2.16	44.88 ± 2.49	52.65 ± 0.79	590.68 ± 8.14	11.59 ± 0.13	11.22 ± 0.19
10.0 ml/kg							
*F*	7605.032	30,430.430	7160.243	518.120	118.773	3433.043	571.931
*ANOVA p-value*	< 0.0001	< 0.0001	< 0.0001	< 0.0001	< 0.0001	< 0.0001	< 0.0001

*V*_*TE*_ expiratory tidal volume, *N.S* not significant, *ΔP* driving pressure, *PIF* peak inspiratory flow, *PEF* peak expiratory flow, *C*_*rs*_ respiratory system compliance, *RC*_*exp*_ expiratory time constant, *R*_*insp*_ inspiratory airway resistance, *R*_*exp*_ expiratory airway resistance

*P values (Student *t* test) are for comparisons between PSV, PCV, and VCV. Data are shown as means ± standard deviation and are the results of eight measurements/case

**Table 3:** Comparisons between VCV (test), PCV, and PSV in the severely obstructive lung model

	ΔP	PIF	PEF	C_rs_	RC_exp_	R_insp_	R_exp_
	(cmH_2_O)	(L/min)	(L/min)	(ml/cmH_2_O)	(ms)	(cmH_2_O/L·s)	(cmH_2_O/L·s)
VCV	8.88 ± 0.05	30.97 ± 0.50	25.04 ± 0.33	50.81 ± 1.28	918.19 ± 15.33	18.07 ± 0.30	21.27 ± 0.30
7.0 ml/kg							
PCV	8.15 ± 0.07	16.06 ± 0.09	16.64 ± 0.16	40.86 ± 1.39	1239.64 ± 9.87	30.37 ± 0.33	30.35 ± 0.44
5.0 ml/kg							
PCV	11.24 ± 0.27	23.38 ± 0.17	23.33 ± 0.51	40.23 ± 0.45	1180.38 ± 15.61	28.83 ± 0.22	29.34 ± 0.29
7.0 ml/kg							
PCV	16.15 ± 0.42	28.92 ± 1.27	27.47 ± 0.53	39.06 ± 0.32	1331.78 ± 14.15	33.47 ± 0.47	34.09 ± 0.38
10.0 ml/kg							
PSV	6.08 ± 0.07	23.75 ± 0.19	15.91 ± 0.17	50.97 ± 1.65^*^	933.50 ± 10.57	15.33 ± 0.18	18.32 ± 0.26
5.0 ml/kg				(*P* = 0.550)			
PSV	9.09 ± 0.13	30.34 ± 1.09	20.30 ± 0.33	45.48 ± 0.61	964.31 ± 10.83	17.96 ± 0.24^*^	21.20 ± 0.32^*^
7.0 ml/kg						(*P* = 0.421)	(*P* = 0.652)
PSV	15.29 ± 0.45	43.73 ± 2.12	29.36 ± 0.55	38.76 ± 0.52	945.45 ± 14.97	20.96 ± 0.20	24.40 ± 0.34
10.0 ml/kg							
*F*	11353.663	11293.037	7544.748	1004.536	1610.687	4818.595	2341.453
*ANOVA p-value*	< 0.0001	< 0.0001	< 0.0001	< 0.0001	< 0.0001	< 0.0001	< 0.0001

*V*_*TE*_ expiratory tidal volume, *N.S* not significant, *ΔP* driving pressure, *PIF* peak inspiratory flow, *PEF* peak expiratory flow, *C*_*rs*_ respiratory system compliance, *RC*_*exp*_ expiratory time constant, *R*_*insp*_ inspiratory airway resistance, *R*_*exp*_ expiratory airway resistance

*P values (Student *t* test) are for comparisons between PSV, PCV, and VCV. Data are shown as means ± standard deviation and are the results of eight measurements/case

**Table 4:** Comparisons between VCV (test), PCV, and PSV in the restrictive lung model

	ΔP	PIF	PEF	C_rs_	RC_exp_	R_insp_	R_exp_
	(cmH_2_O)	(L/min)	(L/min)	(ml/cmH_2_O)	(ms)	(cmH_2_O/L·s)	(cmH_2_O/L·s)
VCV	17.29 ± 0.18	27.29 ± 0.35	79.37 ± 0.54	25.91 ± 0.21	226.23 ± 13.63	8.73 ± 0.49	13.07 ± 0.12
7.0 ml/kg							
PCV	13.56 ± 0.09	32.99 ± 0.86	33.27 ± 1.28	24.02 ± 0.15	450.90 ± 2.68	24.64 ± 0.70	18.77 ± 0.18
5.0 ml/kg							
PCV	18.61 ± 0.17	49.33 ± 0.99	45.00 ± 3.43	24.35 ± 0.25	458.16 ± 4.86	22.62 ± 0.48	19.82 ± 0.12
7.0 ml/kg							
PCV	26.80 ± 1.21	62.09 ± 2.74	54.66 ± 3.73	24.41 ± 0.16	551.27 ± 7.03	25.87 ± 0.28	22.58 ± 0.13
10.0 ml/kg							
PSV	5.46 ± 0.05	49.15 ± 0.12	45.14 ± 1.11	57.59 ± 0.54	295.31 ± 2.87	6.66 ± 0.07	5.47 ± 0.06
5.0 ml/kg							
PSV	11.46 ± 0.09	70.44 ± 1.20	61.74 ± 3.21	36.46 ± 0.25	304.89 ± 0.53	9.75 ± 0.07	8.36 ± 0.06
7.0 ml/kg							
PSV	18.64 ± 1.18	94.54 ± 3.19	79.66 ± 4.35	31.67 ± 0.33	316.04 ± 0.64	11.82 ± 0.10	10.52 ± 0.10
10.0 ml/kg							
*F*	25751.725	11948.118	22445.061	14010.828	3032.487	3647.883	23177.344
*ANOVA p-value*	< 0.0001	< 0.0001	< 0.0001	< 0.0001	< 0.0001	< 0.0001	< 0.0001

*V*_*TE*_ expiratory tidal volume, *N.S* not significant, *ΔP* driving pressure, *PIF* peak inspiratory flow, *PEF* peak expiratory flow, *C*_*rs*_ respiratory system compliance, *RC*_*exp*_ expiratory time constant, *R*_*insp*_ inspiratory airway resistance, *R*_*exp*_ expiratory airway resistance

*P* values (Student *t* test) are for comparisons between PSV, PCV, and VCV. Data are shown as means ± standard deviation and are the results of eight measurements/case

**Table 5:** Comparisons between VCV (test), PCV, and PSV in the mixed obstructive and restrictive lung model

	ΔP	PIF	PEF	C_rs_	RC_exp_	R_insp_	R_exp_
	(cmH_2_O)	(L/min)	(L/min)	(ml/cmH_2_O)	(ms)	(cmH_2_O/L·s)	(cmH_2_O/L·s)
VCV	17.44 ± 0.24	27.61 ± 0.11	45.61 ± 0.34	25.54 ± 0.38	496.51 ± 12.31	19.44 ± 0.25	22.94 ± 0.34
7.0 ml/kg							
PCV	13.37 ± 0.04	23.74 ± 0.31	23.97 ± 0.15	24.22 ± 0.09	646.27 ± 8.22	33.76 ± 0.37	26.68 ± 0.32
5.0 ml/kg							
PCV	18.46 ± 1.16	34.95 ± 0.36	32.89 ± 0.42	24.26 ± 0.20	653.59 ± 7.43	34.66 ± 0.53	26.94 ± 0.46
7.0 ml/kg							
PCV	25.58 ± 2.21	43.77 ± 1.17	42.11 ± 1.20	23.92 ± 0.10	720.08 ± 6.41	35.05 ± 0.88	30.10 ± 0.31
10.0 ml/kg							
PSV	10.24 ± 0.07	29.21 ± 0.06	24.44 ± 0.22	31.02 ± 0.22	585.39 ± 6.42	21.00 ± 0.16	18.87 ± 0.14
5.0 ml/kg							
PSV	15.34 ± 1.09	39.69 ± 0.43	31.60 ± 0.47	26.83 ± 0.22	586.99 ± 5.37	23.16 ± 0.11	21.88 ± 0.30
7.0 ml/kg							
PSV	23.34 ± 2.14	58.75 ± 1.31	46.06 ± 2.33	25.35 ± 0.16*	582.84 ± 6.38	23.81 ± 0.21	23.00 ± 0.29^*^
10.0 ml/kg				(*P* = 0.215)			(*P* = 0.732)
*F*	12318.047	4617.549	8138.969	1056.317	659.218	1737.310	1117.010
*ANOVA p-value*	< 0.0001	< 0.0001	< 0.0001	< 0.0001	< 0.0001	< 0.0001	< 0.0001

*V*_*TE*_ expiratory tidal volume, *N.S* not significant, *ΔP* driving pressure, *PIF* peak inspiratory flow, *PEF* peak expiratory flow, *C*_*rs*_ respiratory system compliance, *RC*_*exp*_ expiratory time constant, *R*_*insp*_ inspiratory airway resistance, *R*_*exp*_ expiratory airway resistance

*P values (Student *t* test) are for comparisons between PSV, PCV, and VCV. Data are shown as means ± standard deviation and are the results of eight measurements/case

### C_rs_ and RC_exp_ at different VT ventilation in the various models

**Fig. 3:**
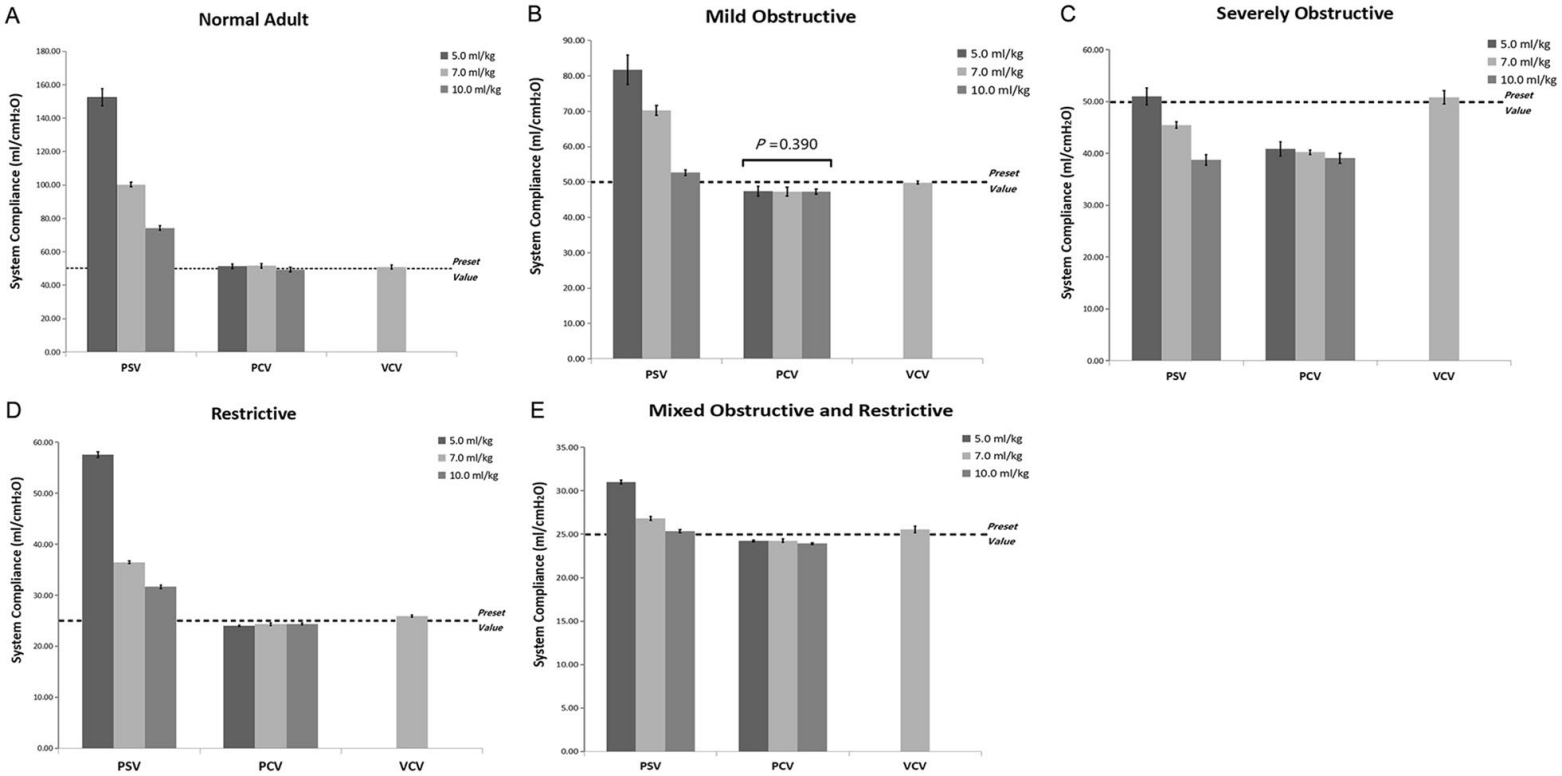
Comparisons of system compliance (C_rs_) in various models during controlled and assisted ventilatory mode. Normal adult (**A**), mildly obstructive (**B**), severely obstructive (**C**), restrictive (**D**), and mixed (**E**) models. Data are presented as mean ± SD. *P* < 0.01 vs. PSV for all pairwise comparisons. The dotted line represents the preset value of C_rs_

C_rs_ and RC_exp_ were calculated according to Eqs. 1 and 2, respectively, as described above, with PEEP kept constant at end-expiration. In the passive breathing conditions, the calculated C_rs_ were close to the preset value, except in the severely obstructive model. When an inspiratory effort was present, the calculated C_rs_ was always overestimated grossly at V_T_ of 5.0–7.0 ml/kg in non-severely obstructive conditions (R_aw_ ≤ 10 cmH_2_O/L•s), and the calculated value was decreased as V_T_ increased (Fig. 3 and Tables 1, 2, and 4).

**Fig. 4:**
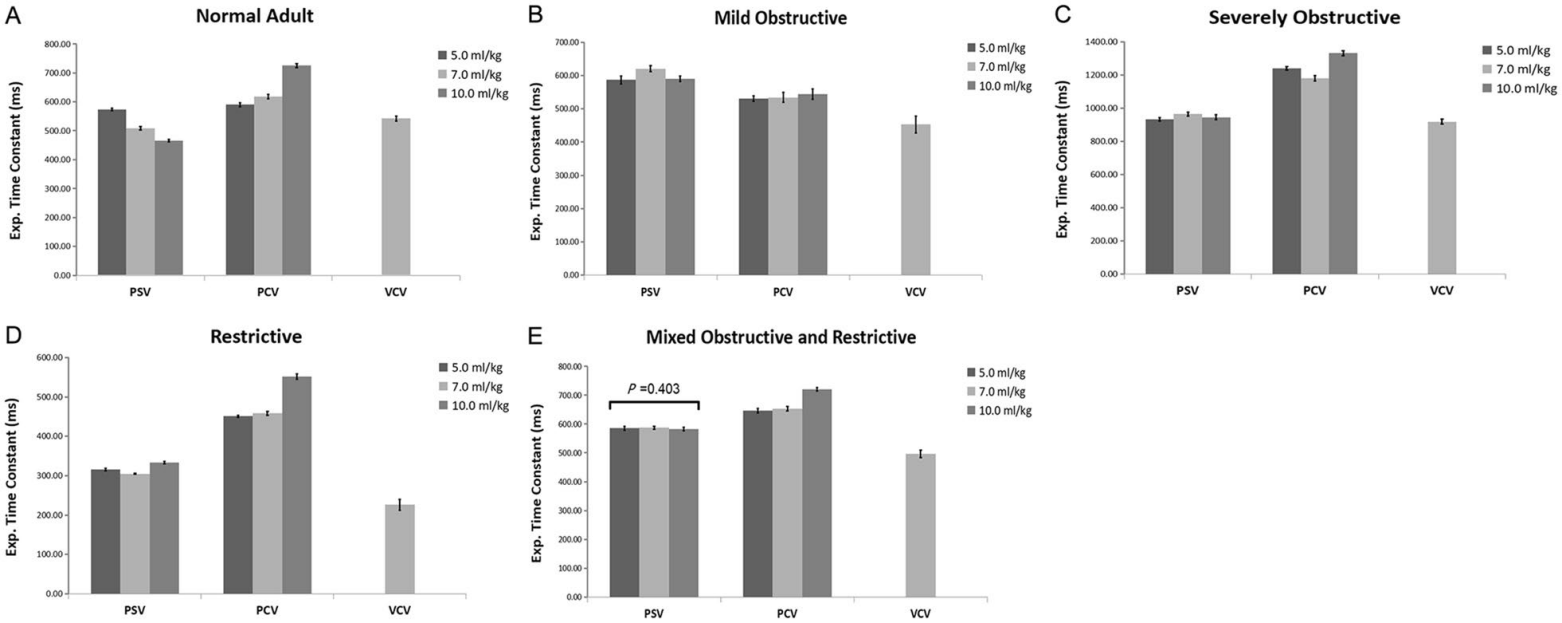
Comparisons of the expiratory time constant (RC_exp_) in various models during controlled and assisted ventilatory mode. Normal adult (**A**), mildly obstructive (**B**), severely obstructive (**C**), restrictive (**D**), and mixed (**E**) models are shown. Data are presented as mean ± SD. *P* < 0.01 vs. PSV for all pairwise comparisons

In the passive breathing condition, the calculation of RC_exp_ in the PCV mode exceeded the value in the VCV mode (*P* < 0.01). During assisted ventilation, the calculated value was slightly affected by V_T_ in all four lung disease models, and no difference was observed at different V_T_ in the mixed obstructive and restrictive model (*P* = 0.403) (Fig. 4).

### Estimated inspiratory and expiratory resistance at different VT ventilation in the various models

**Fig. 5:**
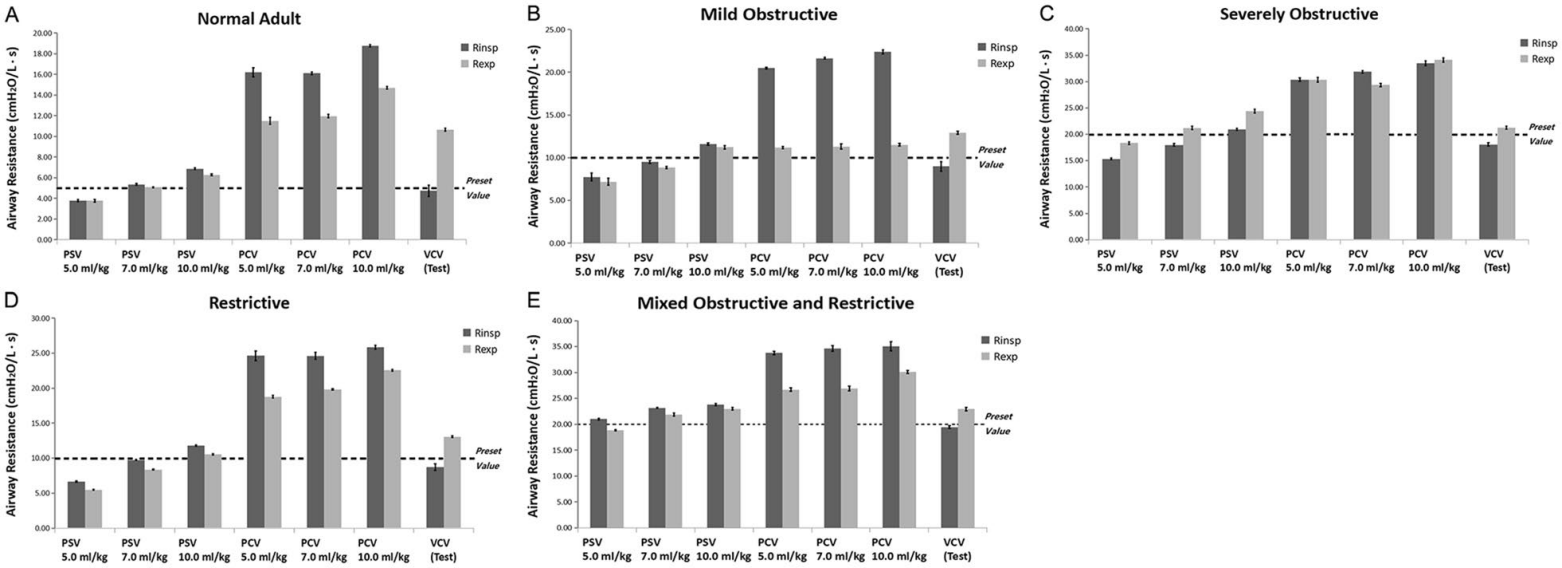
Comparisons of inspiratory (R_insp_) and expiratory resistance (R_exp_) in various models during the controlled and assisted ventilatory mode. Normal adult (**A**), mildly obstructive (**B**), severely obstructive (**C**), restrictive (**D**), and mixed (**E**) models are shown. Data are presented as mean ± SD. *P* < 0.01 vs. PSV for all pairwise comparisons. The dotted line represents the preset value of R_aw_

R_insp_ and R_exp_ were calculated according to Eqs. 3 and 4. During assisted ventilation, R_insp_ was underestimated at V_T_ of 5.0 ml/kg in all five models. The calculated value generally increased with increasing V_T_. The estimated error might be minimal at V_T_ of 7.0 ml/kg, regardless of the change of respiratory mechanics. In passive breathing, R_insp_ was always overestimated despite the alteration of V_T_. Similar results were obtained for calculated R_exp_. The difference between the calculated and preset values was reduced remarkably at V_T_ of 7.0 ml/kg in the PSV model (Fig. 5).

## Discussion

Currently, static measurements are performed at end-inspiration and end-expiration occlusion, as provided by many mechanical ventilators, which is a standard and classic method, and the data represent the static mechanical properties of the respiratory system. The occlusion method should be fulfilled with no flow and a static tidal volume. It is essential that the patient is not allowed to force during static measurements, whether due to disease, sedation, or paralysis [24-26]. Furthermore, it is assumed that C_rs_ is linear between end-inspiration and end-expiration [27, 28]. In reality, during assisted ventilation, an inspiratory effort always exists in spontaneously breathing patients. The change in airway pressure generated by P_mus_ mainly depends on diaphragm activity and the driving pressure output by the ventilator. Since P_mus_ varies with time and among individuals, sampling and analyzing such respiratory system mechanics is very difficult [29].

Dynamic estimations might assess the mechanical characteristics of the respiratory system during assisted ventilation with the variable gas flow. With recent advances in monitoring technology and sophisticated software, real-time estimation at the bedside is a helpful diagnostic tool for assisting therapeutic decisions and adjusting the ventilator settings [9, 30]. The least-square fitting (LSF) technique is one type of dynamic measurements. Recursive least squares (RLS) is a modified LSF technique that derives values for C_rs_ and R_aw_ by solving a linear regression equation in which P_aw_, V_T_, and flow measurements are multiple recorded times (up to 100–200 Hz) during the respiratory cycle [31].

Another dynamic estimation technique is based on RC_exp_ calculation. RC_exp_ contains information about the mechanical properties of the respiratory system (elastance and resistance) and is defined as the product of the total respiratory system compliance and expiratory resistance. RC_exp_ is expressed in units of time (s), and 1 RC_exp_ represents the time required for the respiratory system to reach 63% of its equilibrium value and is an indication of the time required for the lung to empty during exhalation [32, 33]. A study showed that C_rs_, R_insp_, and R_exp_ could be estimated in real-time using RC_exp_ calculation and combined with some equations during mandatory controlled ventilation and assisted ventilation, such as PSV [13]. Čandik et al. [34] observed the relationship between RC_exp_ and breathing cycle time (T_cycle_) during PSV and provided the equation: T_cycle_ = 5.2625 × RC_exp_ + 0.1242(*R*^2^ = 0.85). In this bench study, the pressure and flow datas were obtained by the sensors built in the ASL5000, and off-line analysis using some specical equations. The RC_exp_ was calculated by the volume/flow ratio at 75% of the exhaled V_T_. During pressure support and PCV, the calculated RC_exp_ value varied with tidal volume alteration in all lung models. Only in the severely obstructive condition, the difference of RC_exp_ value between different tidal volume was not significant in the PSV mode (*P* > 0.05). Similarly, the calculations of R_insp_ and R_exp_ were also affected by the V_T_ in all lung conditions. The difference between the calculation and preset value was minimal at V_T_ of 7.0 ml/kg.

During pressure-preset ventilation, such as PSV, the airway pressure waveform is rectangular, and the inspiratory flow varies; the dynamics of lung filling and emptying can be exactly described by exponential equations and is affected by ventilatory parameters and respiratory system characteristics [16]. The advantage of PSV is that the variable inspiratory flow can meet the patient’s demand and improve comfort. PSV must be triggered by the patient’s inspiratory effort. Usually, the patient’s effort is detected by a pressure trigger or a flow trigger. During non-invasive ventilation, the most used trigger mechanism is the flow shape-signal technique, which applies a mathematical model derived from the flow and pressure signals, with better tolerance and reduced trigger asynchrony [14, 35]. The dynamic approach was selected, because it requires neither special maneuvers nor particular flow patterns and does not rely on the amplitude and shape of inspiration effort (P_mus_). In this bench study, the simulator was ventilated in the pressure support mode with exponential decay of inspiratory flow waveform. The driving pressure was calculated as EIP–PEEP, with EIP obtained at the end-inspiration phase after the inspiratory flow is deduced from the PIF. C_rs_ calculation is restricted to the tidal volume and driving pressure [36]. Iotti et al. [37] found that calculated C_rs_ could be affected by the PS levels. With low PS levels and high spontaneous breathing activity, calculated C_rs_ was overestimated, while R_aw_ was underestimated; similar C_rs_ values were obtained at equal V_T_ during PSV with mandatory controlled ventilation (CMV) at a constant flow. In this bench study, the lung simulator was set to simulate an adult with normal body weight (65–70 kg), considering C_rs_ and R_aw_ remained constant throughout any given breath. We demonstrated that the calculated C_rs_ gradually decreased with increasing PS levels and V_T_. With normal–mild obstructive (R_aw_ ≤ 10 cmH_2_O/L/s) and/or strong inhalation effort, the estimation of C_rs_ always exceeded the preset value. This may be due to the patient’s spontaneous effort, rather than to the changes in V_T_. Only in the severely obstructive conditions, the patient’s breathing pattern was dependent upon the ventilator parameters setting, not on the effort. The difference of C_rs_ between the calculated and preset values might be minimal at V_T_ of 7.0 ml/kg.

The present study has some limitations. First, all tests were performed on the ASL 5000 Lung Simulator and under several typical lung mechanics setting. The one-compartment linear model was selected, which assumes that static C_rs_ and R_aw_ remain constant throughout the respiratory cycle. Intrinsic PEEP (auto-PEEP) was also not preset in this study. Nevertheless, it is clear that compliance depends on the volume status, and the resistance is both volume and flow-dependent [38]. The value of compliance throughout the inspiration changes with increasing airway pressure. Stahl et al. [39] found that compliance appears to decline at higher levels of inspiratory pressure during tidal breathing. The quasi-static compliance is increased until the airway pressure reaches 30 cmH_2_O; nevertheless, dynamic compliance is decreased when the airway pressure is above 15 cmH_2_O. Second, resistance changes with the level of flow through the tube on which it is being measured. The higher the flow through the resistive path, the higher the resistance in the path and vice versa. In this way, the information presented to the user represents the maximum resistance experienced by the patient during the phases of the breath. Third, an ICU ventilator (Hamilton C3) was used in this bench study to obtain the quasi-static respiratory mechanics with the occlusion method. During volume-controlled ventilation, the inspiratory flow was kept constant in the inspiration phase, and the circuit was airtight without the mask and accessories. Since the gas flow is always variable and air leaks are found during non-invasive ventilation, the dedicated NPPV ventilators (such as V60 bi-level ventilator) with especially designed electromagnetic valves and leak compensation algorithm exhibit more homogeneous behavior than ICU ventilators on patient–ventilator synchrony [40]. As a bench study, it is unclear if the scheme can be fully translated in a clinical setting such as rapid shallow breathing. Therefore, additional studies are needed to confirm our findings.

## Conclusions

In conclusion, the application of the concept of RC_exp_ to spontaneously breathing subjects is feasible. Using simple calculation equations, the estimation of respiratory mechanics could be accurately and continuously by adjusting the PS levels in spontaneously breathing patients. Different from the occlusion method, monitoring of the RC_exp_ allows assessing the overall respiratory mechanics without interrupting the patient’s breathing flow. The estimated accuracy of the system compliance clearly depends on the volume status and inspiratory effort in spontaneous breathing patients, whereas resistance calculation error might be minimal at a V_T_ of 7.0 ml/kg.

## Data Availability

The data sets used and/or analysed during the current study are available from the corresponding author on reasonable request.

## Abbreviations

NPPV: Non-invasive positive pressure ventilation
PCV: Pressure-controlled ventilation
PSV: Pressure support ventilation
VCV: Volume-controlled ventilation
PEV: Plateau exhalation valve
FIO2: Inspired oxygen fraction
PEEP: Positive end-expiratory pressure
VTE: Expiratory tidal volume
ΔP: Driving pressure
PIF: Peak inspiratory flow
PEF: Peak expiratory flow
TEF75: The flow at 75% of the expiratory VT
Crs: Respiratory system compliance
RCexp: Expiratory time constant
Rinsp: Inspiratory airway resistance
Rexp: Expiratory airway resistance
N.S: Not significant
